# Transfemoral aortic valve replacement in a nonagenarian with aortic stenosis and cardiac amyloidosis: case report

**DOI:** 10.1186/s13019-022-01776-z

**Published:** 2022-03-09

**Authors:** Deena Akras, Keith Bullinger, Meera Kondapaneni, Aisha Siraj, Rami Akhrass, Ashish Aneja

**Affiliations:** 1 Department of Thoracic and Cardiovascular Surgery, Heart, Vascular and Thoracic Institute, Cleveland Clinic, 9500 Euclid Avenue, Cleveland, OH 44195 USA; 2 Department of Cardiology, Heart and Vascular Institute, MetroHealth Medical Center, Cleveland, OH USA

## Abstract

**Background:**

Cardiac amyloidosis (CA) is diagnosed with increasing frequency in the elderly population with severe aortic stenosis (AS), especially with the low-flow, low- gradient phenotype. Prognosis is poor with no treatment.

**Case presentation:**

The patient is a 94-year-old active male who presented with a stroke that fully resolved. He was found to have low-flow, low-gradient severe AS, along with concomitant CA. Gradients across the aortic valve worsened with the dobutamine challenge test. He underwent successful transfemoral aortic valve replacement (TAVR) and did well postoperatively, where he remained in the hospital for only one day. Treatment of his CA with Tafamidis was recommended; however, the patient declined due to its cost and personal preference.

**Conclusion:**

To our knowledge, we report on one of the oldest patients to undergo TAVR for low-flow, low-gradient AS with concurrent CA (AS-CA). It might be prudent to screen elderly patients with AS for CA, as prognosis is worse with medical management alone. TAVR has overall improved survival in patients with AS-CA and is considered the procedure of choice, as these patients are typically older and at higher risk for surgical intervention.

## Introduction

Aortic stenosis (AS) is the most common valvular heart disease, with a prevalence of 4% in patients older than 80 years of age. Cardiac amyloidosis (CA), manifested by extracellular deposit of amyloid fibrils within the myocardium and other structures, affects up to 25% of octogenarians. CA has been increasingly detected and diagnosed due to advances in cardiac imaging, and is reportedly associated with a threefold increase in mortality in patients with AS when left untreated [1, 2].

It is estimated that 15% of patients with AS and up to 30% of the subset with low-flow, low-gradient AS may have concomitant CA, which increases the risk of heart failure and mortality [2]. AS with CA (AS-CA) is associated with significantly worse outcomes for patients who undergo medical management alone, with a reported one-year mortality of 54% compared to 31% for isolated AS. However, with aortic valve replacement (AVR), mortality improves to 16% for AS-CA compared with 10% for isolated AS [3].

Transfemoral aortic valve replacement (TAVR) has become the procedure of choice for severe AS in the elderly who are at higher risk with surgical AVR (SAVR), and its use has steadily expanded to include patients in lower risk groups as well [4]. It has been shown to improve survival compared with medical management in patients with AS associated with CA [3]. We report on one of the oldest patients to our knowledge who underwent TAVR for low-flow, low-gradient severe AS with associated CA. Informed consent was obtained from the patient. Clinical trial registration and institutional review board approval were not required and were waived for the purpose of this study.

## Case report

The patient is a 94-year-old active male with a history of hypertension, hyperlipidemia and stage 3A renal insufficiency, who presented to the emergency department with mental status changes, in addition to months-long worsening of exertional dyspnea. He was diagnosed with a stroke with findings on head computed tomography of a hypodense area in the right occipital region, most likely due to new-onset atrial fibrillation (AF). Echocardiography showed concentric left ventricular wall thickening with left ventricular ejection fraction (LVEF) of 40%, in addition to evidence of severe AS with aortic valve (AV) area of 0.8 cm^2^ and peak and mean gradients of 29 mmHg and 18 mmHg, respectively. Heart catheterization demonstrated mild coronary artery disease as well as low-flow, low-gradient severe AS.

**Fig. 1:**
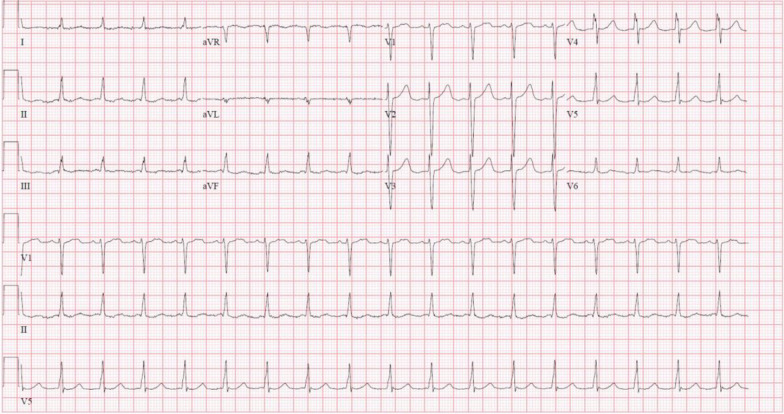
Preoperative electrocardiogram showing lack of LVH criteria

**Fig. 2:**
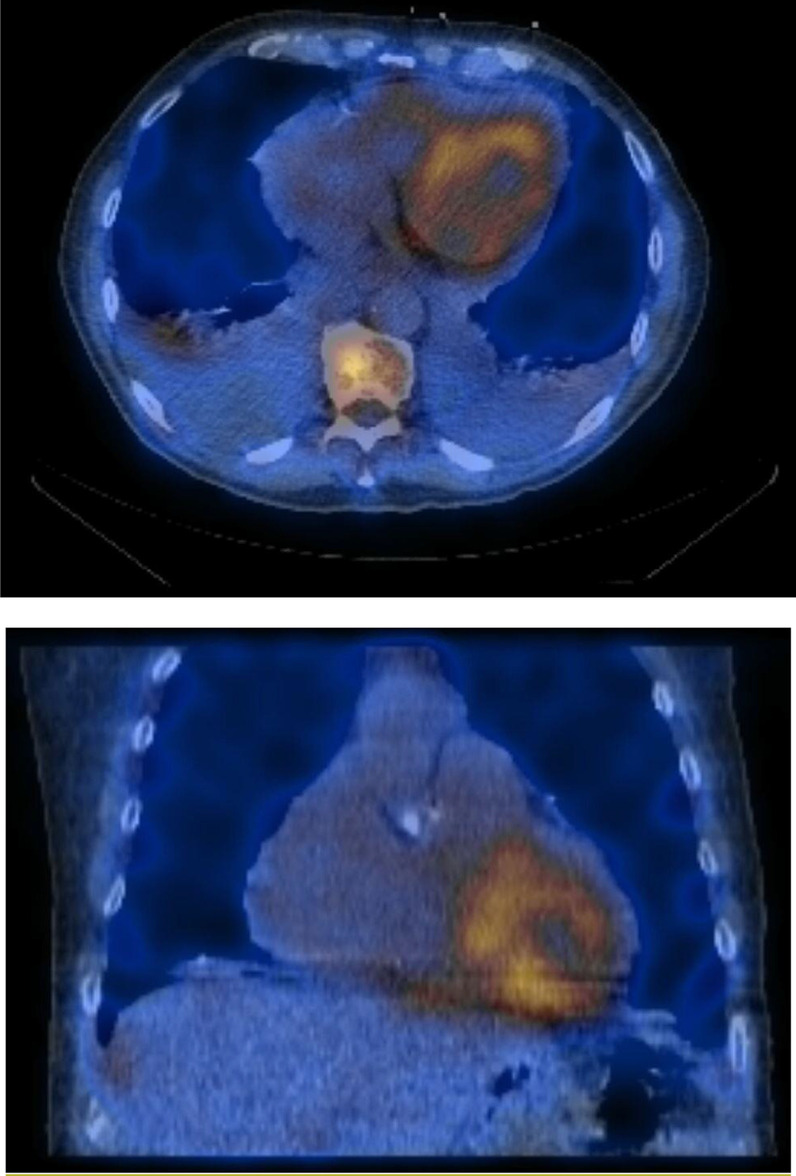
Technetium pyrophosphate (PYP) nuclear scintigraphy showing significant uptake of PYP in the cardiac silhouette

Baseline mean AV gradient was 18 mmHg with a valve area of 0.6 cm^2^ and transvalvular flow rate of 112.18 mL/sec. Dobutamine challenge at 10 mcg/kg/min revealed an increase in mean AV gradient to 47 mmHg and valve area of 0.8 cm^2^. The transvalvular flow rate more than doubled to 243.45 mL/s, confirming severe AS. The patient’s electrocardiogram (Fig. 1) did not show signs of left ventricular hypertrophy (LVH), raising suspicion for CA. Nuclear scintigraphy with technetium pyrophosphate was highly consistent with wild-type (transthyretin) cardiac amyloidosis (Fig. 2).

**Fig. 3:**
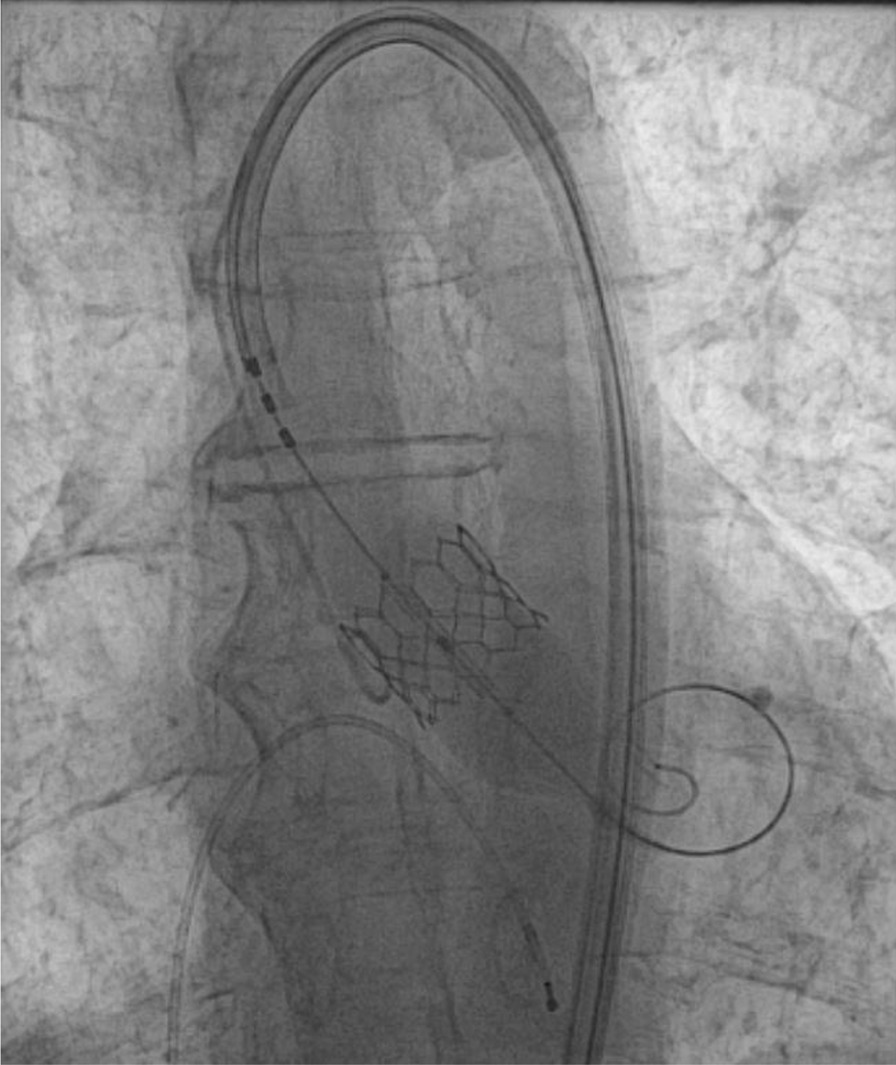
Transfemoral aortic valve replacement (TAVR)

The patient was placed on aspirin (ASA) and direct oral anticoagulant (DOAC) for his AF and stroke. His neurological symptoms completely resolved at the time of his initial presentation, and he underwent successful TAVR two months later (Fig. 3). Postoperatively, the patient was observed in the intensive care unit and was discharged home the following day with no complications. His anticoagulants (ASA and DOAC) were continued, and follow-up echocardiography demonstrated normal functioning of the prosthetic valve with AV area of 2.0 cm^2^ and peak and mean gradients of 9 mmHg and 5 mmHg, respectively. Treatment of his CA with Tafamidis was recommended; however, the patient declined due to its cost and personal preference.

## Discussion

AS and CA are more prevalent in older patients. However, age alone does not explain their frequent concurrence, as the prevalence of CA in the general population older than 80 years of age is only 1% compared with 8–16% observed in patients with AS [5, 6]. CA is a progressive infiltrative cardiomyopathy in which deposits of amyloid, either immunoglobulin light-chain (AL) or transthyretin amyloidosis (ATTR), accumulate in the myocardium. ATTR is usually acquired and of the wild-type, formerly known as senile amyloidosis [7]. ATTR cardiac amyloidosis (ATTR-CA) is the most common cause of restrictive cardiomyopathy in the elderly, and diagnosis is frequently delayed until later stages, when symptoms of heart failure appear. The median survival in untreated patients from the time of diagnosis is around 25–41 months, with longer survival in patients with wild-type CA [6].

AS associated with CA has worse clinical presentation and portends a worse prognosis unless treated [3, 7]. A clinical scoring system (RAISE) has been developed to screen patients with AS for concomitant CA, including: cardiac Remodeling (left ventricular hypertrophy, diastolic dysfunction), Age, cardiac Injury (high-sensitivity troponin T, Systemic disease (carpel tunnel syndrome) and Electrical disturbances (right bundle branch block, low voltage). Such patients should receive further testing with cardiac imaging and free light chain assessment [5].

In patients with symptomatic severe low-flow, low-gradient AS with reduced LVEF, the American College of Cardiology recommends echocardiographic measurements with low-dose dobutamine stress testing if certain criteria on the patient’s baseline echocardiogram are met. These consist of a calcified aortic valve with reduced systolic opening, LVEF less than 50%, calculated valve area 1.0 cm^2^ or less, and aortic velocity less than 4.0 m/s or mean gradient less than 40 mmHg. Severe AS is confirmed if the transvalvular velocity reaches 4.0 m/s with a valve area less than 1.0 cm^2^ during low-dose dobutamine infusion up to a maximum of 20 mcg/kg/min. Stress testing also helps to identify whether or not significant contractile reserve is present. Patients who do not show an increase in stroke volume of at least 20% in response to dobutamine carry a poor prognosis [8]. Unique in our patient is his preservation of contractile reserve, where his aortic transvalvular flow rate more than doubled during a dobutamine 10-mcg/kg/min challenge exam.

The introduction of more effective treatments for ATTR amyloidosis promises to reduce the post-TAVR mortality further in this high-risk group. Chemotherapy directed toward plasma cell dyscrasias in AL amyloidosis can be effective. Tafamidis, a transthyretin-stabilizing benzoxazole derivative, has been shown to reduce mortality [9]. Other medications, known as ATTR silencers, have been approved for the treatment of ATTR associated with neuropathy. These drugs act through RNA interference mechanisms and show promise in preventing myocardial seeding with ATTR as well as chain formation. Investigation into ATTR-CA is rapidly changing the accepted paradigm of a progressive and terminal disease to one that is becoming ever more treatable.

The prevalence of ATTR-CA in patients undergoing TAVR is 8–16%. The presence of ATTR-CA in patients undergoing TAVR was found not to affect post-TAVR mortality; however, an increase in post-TAVR heart failure hospitalizations has been reported and is thought to be secondary to underlying infiltrative cardiomyopathy [6]. Given the high prevalence of AS and increasing frequency of TAVR procedures, it might be prudent to screen patients in whom there is suspicion of associated ATTR-CA, as management and prognosis differ from other cardiomyopathies [10].

## Conclusion

To our knowledge, we report on one of the oldest patients to undergo TAVR for low-flow, low-gradient severe AS associated with CA. The fact that our patient retained significant contractile reserve and achieved an aortic transvalvular flow rate of nearly 250 mL/s is noteworthy. Elderly patients with AS commonly have concomitant CA and further pharmacological challenge testing might be advisable, especially when other associated features are present. Medical treatment alone for AS-CA carries a poor prognosis and valve replacement should be strongly considered. TAVR is advised over SAVR, as these patients are typically older and at higher surgical risk.

## Data Availability

All data generated or analyzed are included in this published article.

## Abbreviations

AF: Atrial fibrillation
AS: Aortic stenosis
AS-CA: Aortic stenosis-cardiac amyloidosis
ATTR: Transthyretin amyloidosis
ATTR-CA: Transthyretin cardiac amyloidosis
AV: Aortic valve
AVR: Aortic valve replacement
CA: Cardiac amyloidosis
LVEF: Left ventricular ejection fraction
SAVR: Surgical aortic valve replacement
TAVR: Transfemoral aortic valve replacement
